# NUQA: Estimating Cancer Spatial and Temporal Heterogeneity and Evolution through Alignment-Free Methods

**DOI:** 10.1093/molbev/msz182

**Published:** 2019-08-19

**Authors:** Aideen C Roddy, Anna Jurek-Loughrey, Jose Souza, Alan Gilmore, Paul G O’Reilly, Alexey Stupnikov, David Gonzalez de Castro, Kevin M Prise, Manuel Salto-Tellez, Darragh G McArt

**Affiliations:** 1 Centre for Cancer Research and Cell Biology, Queen’s University Belfast, Belfast, United Kingdom; 2 School of Electronics, Electrical Engineering and Computer Science, Queen’s University Belfast, Belfast, United Kingdom; 3 Department of Oncology, School of Medicine, Johns Hopkins University, Baltimore, MD

## Abstract

Longitudinal next-generation sequencing of cancer patient samples has enhanced our understanding of the evolution and progression of various cancers. As a result, and due to our increasing knowledge of heterogeneity, such sampling is becoming increasingly common in research and clinical trial sample collections. Traditionally, the evolutionary analysis of these cohorts involves the use of an aligner followed by subsequent stringent downstream analyses. However, this can lead to large levels of information loss due to the vast mutational landscape that characterizes tumor samples.

Here, we propose an alignment-free approach for sequence comparison—a well-established approach in a range of biological applications including typical phylogenetic classification. Such methods could be used to compare information collated in raw sequence files to allow an unsupervised assessment of the evolutionary trajectory of patient genomic profiles.

In order to highlight this utility in cancer research we have applied our alignment-free approach using a previously established metric, Jensen–Shannon divergence, and a metric novel to this area, Hellinger distance, to two longitudinal cancer patient cohorts in glioma and clear cell renal cell carcinoma using our software, NUQA.

We hypothesize that this approach has the potential to reveal novel information about the heterogeneity and evolutionary trajectory of spatiotemporal tumor samples, potentially revealing early events in tumorigenesis and the origins of metastases and recurrences.

***Key words:*** alignment-free, Hellinger distance, exome-seq, evolution, phylogenetics, longitudinal.

## Introduction

Investigating evolution and heterogeneity of a neoplasm can give insight to the nature and origins of therapeutic resistance as well as assist in predicting response to treatment (Greaves and Maley 2012; Turajlic et al. 2018) As a result, and due to the decreasing costs of next-generation sequencing (NGS), there has been a recent increase in longitudinal profiling of patient samples throughout their care leading to a number of high-quality studies (Gerlinger et al. 2014; Johnson et al. 2014; Mazor et al. 2015; Turajlic et al. 2018). However, there are limitations introduced by bulk sequencing of a tumor and a lack of bioinformatic tools to handle these analyses. Phylogenetic reconstruction is commonly used to study evolution in biology, and so, it would be intuitive to apply this to study clonal evolution in cancer (Nowell 1976). However, current studies build phylogenies based on knowledge from only one type of somatic mutation, such as single nucleotide variants (SNVs) and copy number alteration (Gerlinger et al. 2014; Martínez et al. 2015). These methods also require an alignment step to highlight somatic mutations occurring in each sample introducing information loss and bias due to intrinsic issues previously highlighted (Kidd et al. 2010; Rosenfeld et al. 2012; Paten et al. 2017). Similarly, a number of methods have been highlighted previously to measure intratumoral heterogeneity (ITH) including the use of ecology measures of diversity in Barrett’s esophagus, the MEDICC algorithm, PyClone, and EXPANDS (Martínez et al. 2015; Schwarz et al. 2015; Andor et al. 2016). However, similar limitations apply here as only one type of somatic alteration is incorporated, such as allele frequency, also requiring the use of an aligner. Additionally, ecological measures, such as identifying the number of clones, can be found relatively easily in “2D” tumors such as Barrett’s esophagus but this would be difficult to replicate in 3D tumors.

Alignment-free sequence comparison, defined as any approach calculating similarity/dissimilarity between sequences which does not use or produce alignment, can be used as an alternative approach to address these issues and create holistic patient profiles for assessing evolutionary trajectories and spatiotemporal heterogeneity. It is more sensitive in the context of sequence divergences and robust against genome rearrangement compared with alignment approaches (Vinga 2014; Bernard et al. 2017). These methods can broadly be split into two groups: word-based methods and information-theory based methods. Here, we will focus on word-based methods which have recently been shown to have greater accuracy compared with information-theory based methods in protein sequence comparison (Zielezinski et al. 2017). The natural efficiency and accuracy of this algorithm has led to its use in many areas including assessing phylogenetic relationships between bacterial and viral genomes, promoter recognition, and protein sequence comparison expanding to an extensive list of tools currently available for various applications (Sims et al. 2009; Chattopadhyay et al. 2015; Fan et al. 2015; Xu et al. 2016), which has been reviewed previously (Zielezinski et al. 2017). However, very few tools can scale to handle the quantity of data as required by longitudinal cancer research cohorts.

Here, we present NUQA (*N*GS tool for *U*nsupervised analysis of fast*Q* using *A*lignment-free), a framework that utilizes a highly efficient *k*-mer counter, jellyfish, alongside software built in C++ to quickly and efficiently produce alignment-free “phylogenetic” trees for longitudinal cancer patient cohorts on a standard workstation. In order to ensure this approach is robustly applicable to cancer research cohorts we have assessed a well-known metric, Jensen–Shannon divergence (JSD), which has previously been applied in an alignment-free context (Sims et al. 2009), as well as a novel metric in this space, Hellinger distance (HD).

## New Approaches

NUQA was developed using bespoke scripts written in bash and C++ along with prebuilt software jellyfish (Marçais and Kingsford 2011) and PHYLIP (Felsenstein 2004). This algorithm consists of five steps: *k*-mer counting using jellyfish; sorting the resulting count vectors for easier processing and normalizing to values between one and zero for comparison; merging the count vectors into a single data matrix using a C++ script; calculating the distances between these vectors using a bespoke C++ script; and finally, building a newick tree using PHYLIP. These steps are combined in a single wrapper script written in bash (supplementary fig. S1, Supplementary Material online). We have tested both JSD and HD for applicability in the comparison of whole-exome sequencing (WES) samples in longitudinal cancer patient cohorts. Given two probability vectors, *P* and *Q*, JSD is defined as:

$$\mathrm{JS}\left(P,Q\right)=\frac{1}{2}KL\left(P,M\right)+\frac{1}{2}KL\left(Q,M\right),$$

where $M=\frac{1}{2}\left(P+Q\right)$ and KL is Kullback–Leibler divergence:

$$\mathrm{KL}\left(P,M\right)=\sum_{i=1}^{k}p_{i}\log_{2}\frac{p_{i}}{m_{i}}.$$

HD is defined as:

$$H\left(P,Q\right)=\frac{1}{\sqrt{2}}\sqrt{\sum_{i=1}^{k}(\sqrt{p_{i}}-\sqrt{q_{i}}{)}^{2}}.$$

Detailed methods are available in the Supplementary Material online (Section 1) and the software implementation can be found on GitHub (https://github.com/ACRoddy/NUQA; last accessed August 22, 2019).

## Results

### Identifying Optimal Parameters

**Fig. 1.**
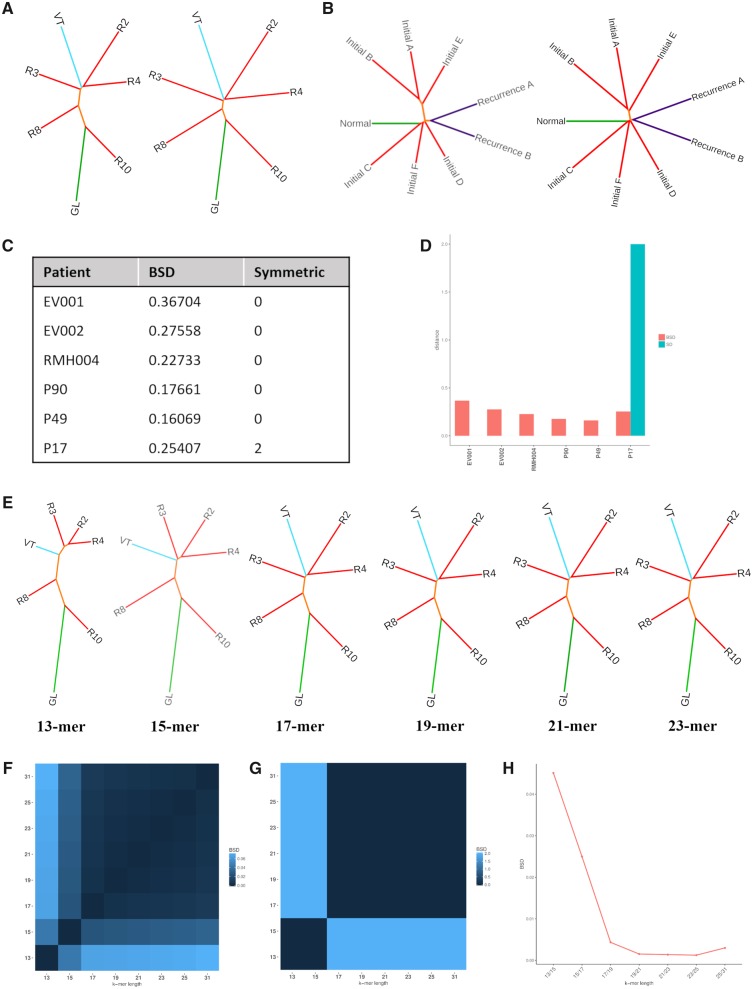
Identifying optimal parameters for use with alignment-free. Application of Jensen–Shannon divergence (JSD) and Hellinger Distance (HD) to (*A*) clear cell renal cell carcinoma (ccRCC) patient RMH004 with a germline sample (GL), multiple samples from the ccRCC tumor (R2-4, R8, R10) and a tumor thrombus from the renal vein (VT) and (*B*) glioma patient P90 with a germline sample (Normal), multiple samples from the initial grade II glioma (Initials A–F) and two samples from a recurrent grade II glioma (Recur 1A and 1B). (*C*) A table summarizing branch-score distance (BSD) and symmetric distance (SD) values returned when comparing trees for six patients for which both JSD and HD have been applied. (*D*) A bar chart summarizing BSD and SD values returned when comparing trees for six patients for which both JSD and HD have been applied. (*E*) Tree topologies produced using *k*-mer lengths 13, 15, 17, 19, 21, and 23 in combination with JSD when applying alignment-free methods to patient RMH004. (*F*) A heatmap representing the BSD between trees produced using varying *k*-mer lengths and HD applied to patient RMH004. (*G*) A heatmap representing the BSD between trees produced using varying *k*-mer lengths and JSD applied to patient RMH004. (*H*) A line graph representing the BSD between trees produced using increasing *k*-mer lengths when applying JSD.

Multiple distance metrics have been highlighted for their utility in alignment-free sequence comparison in various studies and reviews (Höhl et al. 2006; Dai et al. 2008; Vinga 2014; Zielezinski et al. 2017), From these we selected the most applicable to our cohort (discussed in the supplementary note 1.2, Supplementary Material online). We decided to focus on JSD, a previously studied metric in alignment-free methods, and HD which is novel to this domain. We applied each of these metrics to 6 patients, 3 clear cell renal cell carcinoma (ccRCC) patients and 3 glioma patients, using a 21-mer length in order to assess their applicability to cancer patient cohorts (fig. 1*A*–*D* and supplementary fig. S2, Supplementary Material online). We compared the trees using both branch-score distance (BSD) and symmetric distance (SD) (fig. 1*C* and *D*). BSD suggests that HD produces similar results to JSD with distances <0.3 for 5/6 patients, whereas SD highlighted that JSD and HD produce the same tree topologies (SD = 0) for all patients except P17 which obtained a SD of 2 due to a change in location of sample “Recurrence A.” We conclude that JSD and HD both produce consistent results in this context suggesting that HD may perform well in other alignment-free applications.

With the aim of identifying an appropriate *k*-mer length which should be used when applying alignment-free methods to longitudinal cancer patient cohorts, we assessed the effect of varying *k*-mer length (13, 15, 17, 19, 21, 23, 25, and 31 bp) for patient RMH004 (fig. 1*E*–*G*) and additionally for patients P90, P17, EV001, and EV002 (supplementary figs. S3–S6, Supplementary Material online, respectively) using JSD. An ideal *k*-mer length would have the sensitivity of representing only one mutation while also ensuring it does not occur frequently or represent multiple regions (supplementary note 1.2, Supplementary Material online).

Again, we compared trees using BSD and SD. Results were visualized using heatmaps (fig. 1*F* and *G*) and a line graph depicting the effects of sequential increases in *k*-mer length on BSD (fig. 1*H*). Results indicate an optimal range of 17–25 for these patients supporting previous findings that 21 is an optimal *k*-mer length for large genomes (Sims et al. 2009; Fan et al. 2015).

### Application to Cancer Patient Cohorts

**Fig. 2.**
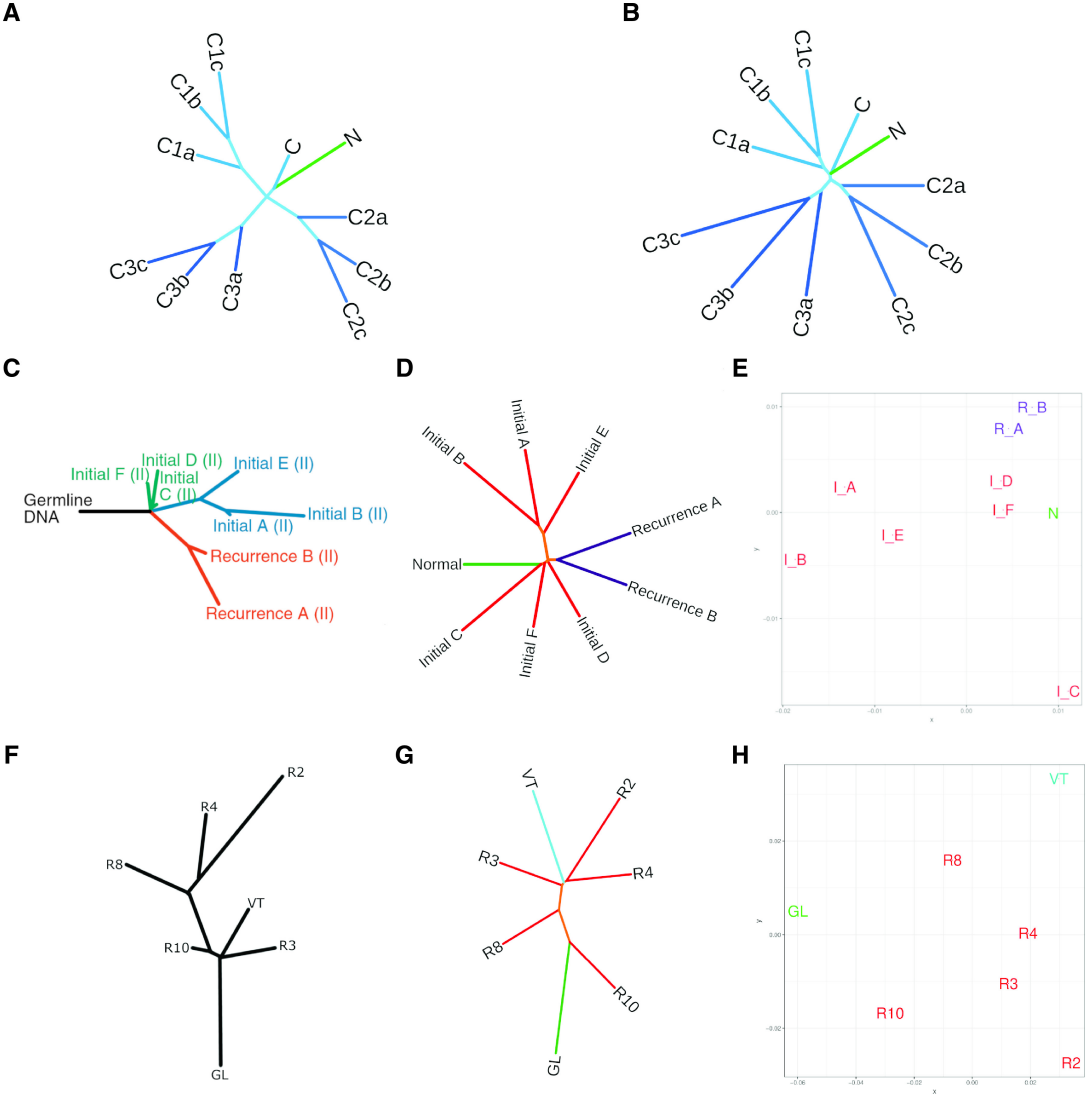
Applying alignment-free sequence comparison methods to glioma patient P90 and ccRCC patient RMH004. (*A*) Simulated data set “A” created using software XS and fastx-mutate-tools to represent SNVs and indels in small scale data such as WES. (*B*) Simulated data set “B” created using software pIRS to represent SNVs and indels and structural variants in WGS. (*C*) Least-square minimum-evolution tree produced based on a binary matrix of SNVs present in the samples for P90 reused with permisson from Mazor et al. (2015). (*D*) An unrooted Neighbor-Joining tree produced applying our alignment-free software (NUQA), incorporating JSD, to patient P90. (*E*) Multidimensional scaling plot representing the distances between samples produced applying NUQA, incorporating JSD, to patient P90. (*F*) A maximum parsimony tree produced based on a binary matrix of SNVs present in the samples for RMH004 adapted with permission from Gerlinger et al. (2014). (*G*) An unrooted Neighbor-Joining tree produced applying NUQA, incorporating JSD, to patient RMH004. (*H*) Multidimensional scaling plot representing the distances between samples produced applying NUQA, incorporating JSD, to patient RMH004.

To first validate the use of this method on longitudinal, spatial, and temporal cohorts, we created simulated data sets, A and B, to represent cancer patient profiles through introducing controlled mutational events (fig. 2*A* and *B*, respectively). The aim was to anticipate a predefined branching pattern and assess the ability of NUQA to correctly assign a branching pattern. A “normal” file (N) was produced initially before being mutated to form a “cancerous” file (C). This cancerous sample was then mutated three separate times to represent heterogeneity (files C1a, C2a, and C3a) and finally each of these three files were mutated two successive times (files b and c) to represent the evolution of these three subclones. Data set A was simulated to represent SNVs and indels within WES data whereas data set B represents SNVs, indels, and structural variants within whole-genome sequencing (WGS) data. As expected, these three subclones form three distinct branches with file “c” being the most distal sample and file “a” being the least.

Furthermore, we identified two well-studied, high-quality longitudinal cancer research cohorts to test the utility of our software in glioma (Johnson et al. 2014; Mazor et al. 2015) and ccRCC (Gerlinger et al. 2014). We have identified one patient from each cohort for whom the original authors have produced phylogenetic trees drawn from the information obtained using a variant caller to highlight SNVs and small indels. Patient P90 from the glioma cohort had longitudinal samples, whole-exome sequenced, including circulating blood samples (Normal), six samples from the initial tumor (Initials A–F) classified as a grade II glioma and two samples from a recurrence tumor (Recur A and B) also classified as a grade II glioma. We applied our own algorithm to this patient and produced phylogenetic trees and MDS plots based on our output (fig. 2*B* and *C*). A least-squares minimum-evolution (LSME) tree was produced from somatic SNVs and indels for patient P90 by Mazor et al., for which, detailed methods can be found in the original paper (Mazor et al. 2015) (fig. 2*A*). We use these as a basis for comparison, aware that bias will have been introduced as only reads which uniquely aligned to the reference genome have been considered and the variant callers used could only identify SNVs and small indels but not larger aberrations. This tree contains a relatively long trunk region before tumor samples diverge indicating linear evolution. Furthermore, three key clusters of samples are formed, the first containing Initials C, D, and F, the second containing Initials A, B, and E and the final cluster containing the two recurrent samples. Similarly, the tree produced using NUQA is highly consistent, also indicating that initial samples C, D, and F occur early in evolution, clustering closely with the Normal sample whereas initial samples A, B, and E branch distally suggesting that these are later events in evolution. In addition, recurrence samples A and B branch early, clustering closely with initial samples C, D, and F. Moreover, both trees seem to suggest high levels of ITH within the initial tumor and that there is little ITH within the recurrent tumor.

For ccRCC patient RMH004 we have WES data for germline DNA in the blood (GL), five samples from the initial ccRCC tumor (R2-4, R8, and R10) and one sample from a thrombus in a renal vein (VT). Again, we produced phylogenetic trees and MDS plots based on our output from NUQA for this patient (fig. 2*E* and *F*). Maximum parsimony trees were created based on SNVs and small indels found to be present within the tumor samples as described in the original paper (Gerlinger et al. 2014) (fig. 2*D*). The original maximum parsimony tree suggests that R3, VT, and R10 occur early in evolution whereas R8, R4, and R2 occur much later and are more highly mutated. The original authors highlighted that two distinct mutations occurred in *PBRM1* indicating parallel evolution of two subclones within the tumor. The phylogenetic tree produced using NUQA also suggests that sample R10 occurs early in evolution and that R2 and R4 are more genetically divergent, occurring much later in evolution (fig. 2*E*). However, samples R3, VT, and R8 show variations in branching suggesting that more complex mutational events may be present in these samples. Both trees also appear to show high levels of ITH which can also be seen in the MDS plot for these samples (fig. 2*F*).

Further analysis of patients P17 and EV001 also indicate similar groupings to what can be seen using alignment-based methods, however, again there are key differences in branching within these patients (supplementary fig. S6; supplementary note 3.1 and fig. S7; supplementary note 3.2, Supplementary Material online, respectively). Additional analyses can be performed based on these results, for example, by using the branching pattern produced through NUQA to inform groups as a basis for further analysis. An example using FastGT (Pajuste et al. 2017) to identify SNP calls differentiating groups found in patient P90 can be found in the supplementary note 3.3 (Supplementary Material online).

### Benchmarking Alternative Alignment-Free Packages

**Fig. 3.**
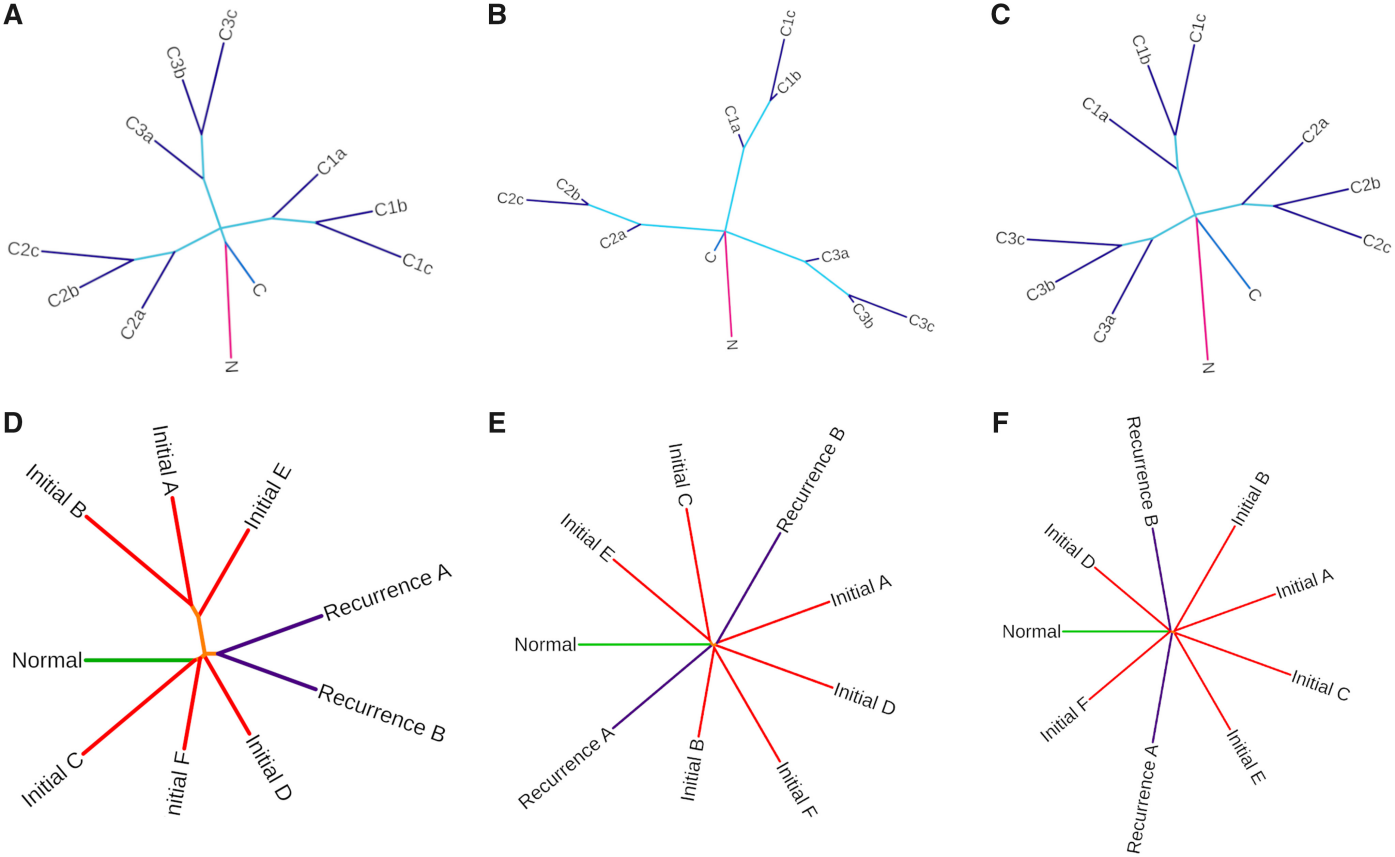
Benchmarking of NUQA against other alignment-free softwares. Unrooted Neighbor-Joining trees produced when applying NUQA (*A*), AAF (*B*), and kWIP (*C*) to a simulated data set using a *k*-mer length of 17 and allowing 64 GB RAM and trees produced when applying NUQA (*D*), AAF (*E*), and kWIP (*F*) to patient P90 using a *k*-mer length of 21 and allowing 64 GB RAM.

Reviewing the literature on current alignment-free phylogenetic software identified two capable of processing multiple large fastq files for sequence comparison: AAF and kWIP (Fan et al. 2015; Murray et al. 2017) both of which are designed to classify organisms at species level requiring a sensitivity to much larger genetic distances. All packages were tested using patient P90 using a *k*-mer length of 21 and allowing 64 GB RAM. AAF produced the best time of 1 h, 57 min whereas NUQA ran in 2 h, 25 min and kWIP ran in 5 h, 48 min. In order to assess the applicability of these to cancer research data we tested NUQA, AAF, and kWIP on our simulated data set (fig. 3*A*–*C*, respectively). It is promising to see that all softwares produce the branching pattern we expect to see. However, when applied to patient P90 (fig. 3*D*–*F*, respectively) we see a variation in tree topology, but more importantly, AAF and kWIP produce a very small trunk (orange) compared with branch lengths indicating that they are less sensitive to the changes occurring between single-patient samples.

## Discussion

Alignment-free sequence comparison is capable of building evolutionary relationships between samples without the use of an aligner. This approach allows for the inclusion of all information regardless of whether it would align to a reference genome preventing bias to pipeline specific information and allowing the inclusion of larger insertions and deletions or chromosomal rearrangements which would be difficult to align. It is also a highly efficient approach yielding grossly improved times over traditional methods using an aligner (Zielezinski et al. 2017).

Here, we have shown potential utility for this approach to be applied to longitudinal cancer patient cohorts as an unsupervised approach for comparing sequencing files. In order to do this we have tested a range of suitable distance metrics for their applicability to this type of data, highlighting JSD as an appropriate measure to assess pairwise distances between feature frequency profiles as previously described (Sims et al. 2009). But also HD, a previously untested metric in alignment-free sequence comparison which we have shown produces equally consistent results. Varying *k-*mer length revealed that a *k*-mer greater than 17 should be sufficient for this analysis, however, we decided to continue further analysis with a *k*-mer length of 21 to reduce the effects of homoplasy. We validated the use of NUQA on longitudinal, spatial, and temporal cohorts using two simulated data sets A and B, representing SNVs and indels in small scale data and SNVs, indels, and structural variants in large scale, WGS data, respectively. Furthermore, we assessed the utility of applying an alignment-free framework in cancer research by applying this method to one patient each from two high-quality longitudinal cohorts in ccRCC (Gerlinger et al. 2014) and glioma (Johnson et al. 2014; Mazor et al. 2015). In both cases, clear similarities could be seen when comparing the results of alignment-free analysis to the trees produced using alignment-based approaches, deduced from changes in SNVs and small indels, however, clear and possibly fundamental differences could be seen. This may be a result of unassessed gene fusion events, larger indels or chromosomal rearrangements which are also contributing to the tumors mutational landscape and therefore affecting the evolutionary pathway of these cancer patients. Finally, we benchmarked our software, NUQA, against other large-scale alignment-free softwares designed for assessing a much greater genetic divergence between samples: kWIP and AAF. We found that AAF yielded a marginal improved speed over our current approach; however, neither software was designed to assess the relatively small genetic distances which would be seen in a cancer patient cohort.

Our tool, in combination with alignment-free genotyping tools, such as FastGT (Pajuste et al. 2017), has the potential to add extra layers to the evolutionary analyses of cancer types providing insights which may otherwise be passed over. Further analysis of the feature frequency profiles built in our extendable alignment-free framework could highlight patterns and abnormalities contributing to the branching pattern obtained for each cancer patient helping to tease out contributing factors in cancer evolution. We would expect that given current precision medicine paradigms and reductions in sequencing costs this approach may be adopted clinically to highlight a cancer trajectory and consequential strategies for the patient.

In conclusion, we have introduced NUQA, a novel and efficient software application for performing alignment-free sequencing comparison, with the aim of highlighting the utility of these methods for the unsupervised phylogenetic assessment of longitudinal patient cohorts in cancer research. We hypothesize that this presents an opportunity to provide a landscape view to identify early and late events in evolution as well as give an indication of the origins of metastatic and recurrent tumors in quick turnaround time and can be used in combination with the more targeted and previously adopted approaches.

## Materials and Methods

This framework was applied to two previous published data sets: A glioma cohort containing spatial and temporal exome-seq data for patients P17, P49, and P90 (Johnson et al. 2014; Mazor et al. 2015), A ccRCC cohort containing spatial and temporal exome-seq data for patients EV001, EV002, and RMH004 (Gerlinger et al. 2014).

Both the glioma and the ccRCC cohort were preprocessed using the same steps prior to applying our algorithm: SAMTOOLs (Li and Durbin 2009) was used to revert files for patient’s P17, EV001, EV002, and RMH004 from bam to fastq files to allow us to work with the raw reads obtained from sequencing. Following this, FastQC (Andrews 2010) was used to ensure the files were a good quality for alignment-free processing and for setting levels for trimming, if required reads were trimmed using Trimmomatic (Bolger et al. 2014). Finally, resulting trees were visualized using the online software tool, iTOL (https://itol.embl.de/). MDS plots were created using the *cmdscale()* function and *ggplot2* (Wickham 2009) package within the R statistical environment (R Core Team 2017).

To assess changes in tree topology and branch lengths between trees produced using alignment-free methods for the same patient we used BSD, a measure accounting for both branch length and tree topology, and SD, a measure accounting for only tree topology. Both of these are available through the PHYLIP package.

Further description of the generation of simulated data and discussion on the choice of distance metric and evaluation of *k*-mer length are available in supplementary notes 1.2–1.6 (Supplementary Material online).

## Supplementary Material

Supplementary data are available at *Molecular Biology and Evolution* online.

## Supplementary Material

msz182_Supplementary_Data
