# Supplementary material for: NUQA: Estimating Cancer Spatial and Temporal Heterogeneity and Evolution through Alignment-Free Methods
Source: Mol Biol Evol. 2019 Aug 19;36(12):2883–9. doi: 10.1093/molbev/msz182 (PMC6878956; doi:10.1093/molbev/msz182)
Supplement: msz182_Supplementary_Data [file msz182_supplementary_data.docx]

Supplementary Materials for

**NUQA: Estimating cancer spatial and temporal heterogeneity and evolution through alignment-free methods**

AC Roddy^1^, A Jurek^2^, J Souza^1^, A Gilmore^1^, PG O’Reilly^1^, A Stupnikov^1, 3^, D Gonzalez de Castro^1^, KM Prise^1^, M Salto-Tellez^1^, DG McArt^1^**

1. Centre for Cancer Research and Cell Biology, Queen’s University Belfast, Belfast, UK

2. School of Electronics, Electrical Engineering and Computer Science, Queen’s University Belfast, Belfast, UK

3. Department of Oncology, School of Medicine, John Hopkins University, Baltimore, USA, MD 21287

****Corresponding author:**

Dr. Darragh G. McArt

Bioinformatics Group, Health Sciences Building,

Centre for Cancer Research and Cell Biology,

Queen’s University Belfast,

97 Lisburn Road, Belfast, BT9 7BL, UK.

Ph: 0044 (0) 28 9097 2629

Email: [d.mcart@qub.ac.uk](mailto:d.mcart@qub.ac.uk)

Table of Contents

**1. Methods3**

Supplementary Note 1.1: Detailed description of the alignment-free implementation 3

Supplementary Note 1.2: Identifying appropriate distance metrics 4

Supplementary Figure S16

Supplementary Note 1.2: Choosing k-mer length 7

Supplementary Note 1.3: Justification of application to cancer research cohorts 8

Supplementary Note 1.4: Data acquisition 9

Supplementary Note 1.5: Data preprocessing 10

Supplementary Note 1.6: Generation of simulated data 11

Supplementary Note 1.7: FastGT analysis 12

**2. Identifying optimal parameters13**

Supplementary Figure S2 13

Supplementary Figure S315

Supplementary Figure S4 16

Supplementary Figure S517

Supplementary Figure S618

**3. Application to cancer patient cohorts19**

Supplementary Figure S7 19

Supplementary Note 3.1: Interpretation of application of alignment-free to P1720

Supplementary Figure S8 22

Supplementary Note 3.2: Interpretation of application of alignment-free to EV00123

Supplementary Note 3.3: Identifying contributions to branching using FastGT24

Supplementary Table 3.1 25

Supplementary Table 3.226

**4. Comparison of NUQA and aligned methods27**

Supplementary Note 4.127

Supplementary Figure S9 28

**References29**

**1. Methods**

**Supplementary Note 1.1: Detailed description of the alignment-free implementation**

NUQA reads in a user-specified list of fastq files for feature extraction by *jellyfish*(Marçais and Kingsford 2011), a memory-efficient *k*-mer counter for DNA sequencing data. *Jellyfish* software increases parallelism by utilizing the “compare and swap” CPU instruction and utilises an efficiently encoded hash table. This can be catered for as single-end or paired-end reads. Using a 2-pass method in *jellyfish* allows for the accurate counting of only high-frequency *k*-mers. There is an initial scan of the data to check its contents and only elements which have been seen at least twice will be included in the hash table which is used for *k*-mer counting. Due to the scale of the fastq files produced by sequencers for tumour samples, low-frequency *k*-mers (*k*-mers with a count of 1) are taken to be unreliable and likely to be a result of sequencing errors(Fan et al. 2015). This allows for the easy removal of low-frequency reads as well as allowing for more time and memory efficient data processing. This produces a file which maps each *k*-mer to a count value for each fastq file supplied. These files are sorted to allow for efficient merging using the command *nuqa_merge*. Given a frequency distribution of *k*-mers for each sample, each distribution is normalised to a value between 1 and 0 (*count/total*) and a distance metric is applied to highlight differences between sample files using command *nuqa_distance*.

**Supplementary Note 1.2: Identifying appropriate distance metrics**

A number of studies have benchmarked a wide range of distance metrics commonly used in AF sequence comparison including Euclidean distance, d2 statistic, Kullback-Leibler discrepancy, linear correlation coefficient and Mahalanobis distance(Vinga et al. 2004; Höhl et al. 2007; Dai et al. 2008; Zielezinski et al. 2017), however, these were benchmarked using relatively low dimensional data and small k-mer windows and therefore cannot be directly transposed for our application. Our count matrix is built using a small number of whole-exome sequencing files with a relatively large k-mer length (k=21) resulting in relatively sparse, high-dimensional data. As a result, we discounted measures of absolute distance such as Euclidean, d2 statistic and Mahalanobis distance as these are generally sensitive to large distances which are common in this type of data and are highly dependent on scale. In contrast, metrics such as JSD and HD emphasise differences between profiles rather than the absolute distances that may skew results by assessing distances on a *k*-mer by *k*-mer basis. With this in mind, we have tested both JSD and HD for applicability in the comparison of WES samples in longitudinal cancer patient cohorts. Given two probability vectors, *P* and *Q,* JSD is defined as:

$$JS\left( P,Q \right)=\frac{1}{2}KL\left( P,M \right)+\frac{1}{2}KL\left( Q,M \right)$$

where $M=\frac{1}{2}\left( P+Q \right)$ and KL is Kullback-Leibler divergence:

$$KL\left( P,M \right)=\sum_{i=1}^{k} p_{i}{log}_{2}\frac{p_{i}}{m_{i}}$$

HD is defined as:

$$H\left( P,Q \right)=\frac{1}{\sqrt{2}}\sqrt{\sum_{i=1}^{k} (\sqrt{p_{i}}-\sqrt{q_{i}})^{2}}$$

**
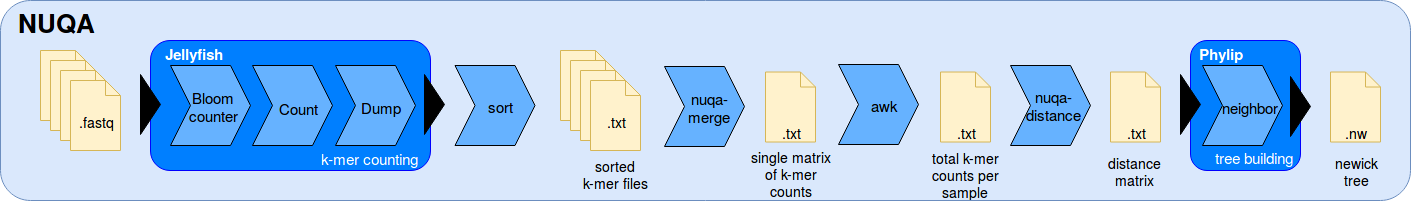
**

**Supplementary Figure S1: Flow diagram for NUQA (NGS tool for Unsupervised analysis of fastQ using Alignment-free).** Our implementation of a k-mer-counting alignment-free algorithm using Jensen-Shannon Divergence and Hellinger distance to measure the distance between k-mer frequency profiles obtained from raw sequencing files.

**Supplementary Note 1.2: Choosing k-mer length**

Choosing a *k*-mer length has been discussed in depth by both Sims *et al.* and Fan *et al.*(Sims et al. 2009; Fan et al. 2015) with the aim of balancing both *k*-mer sensitivity and homoplasy. A sensitive *k*-mer length aims to represent a mutation by only one *k*-mer and avoiding having a *k*-mer length which may contain multiple mutations. However, if *k* is too small it will occur frequently across samples causing homoplasy, this can be overcome by increasing *k*. Thus, the aim is to obtain an optimal *k* which is just large enough to overcome homoplasy given a genome size. Sims *et al*. suggest using the following formula as a minimum *k*-mer length:

$$K_{min}={log}_{4}\left( n \right)$$

where 4 is the alphabet size, {A, T, C, G}, and n is genome length. *k* should then be increased until topology is consistent. Multiple authors have taken a similar approach by assessing the effect of increasing *k* and found that no improvement was obtained by increasing *k* beyond 21(Aflitos et al. 2015; Fan et al. 2015). The effect of varying *k*-mer length (13, 15, 17, 19, 21, 23, 25, 31bp) was also assessed using a test case in this body of work.

**Supplementary Note 1.3: Justification of application of cancer research cohorts**

Our aim is to elucidate new information from cancer cohorts by incorporating more information than current analysis pipelines allow. Therefore, it is difficult to highlight what is the ‘correct’ phylogenetic output. However, through our pre-processing we aim to remove any poor quality reads using trimmomatic and remove low frequency *k*-mers (which may be sequencing artefacts) using *jellyfish*. Our aim, through this is that we are processing only information which represents the sample and therefore, due to strongly supported studies in alignment-free, the information we supply to the methodology is as optimal as possible.

**Supplementary Note 1.4: Data acquisition**

Original BAM files were obtained for ccRCC patients EV001, EV002 and RMH004 from the European Genome-phenome Archive under accession code EGAS00001000667.

Original BAM files were obtained for glioma patients P17, P49 and P90 from the European Genome-phenome Archive under accession codes EGAS00001001255 and EGAS00001000579.

**Supplementary Note 1.5: Data preprocessing**

For the purposes of showing the utility of NUQA as a framework to elucidate evolutionary patterns within longitudinal cohorts we have decided to clean the data by trimming reads based on quality and removing low frequency k-mers as described in the methods section. Further steps which some users may want to use when preprocessing data include deduplication and removal of contaminants. To address the issue of removing duplicate reads from a fastq file we have developed an alignment-free deduplication method utilizing apache pig which is available on GitHub (<https://github.com/joselrsousa/dedup>). In addition, contaminants can be identified using K-mer Analysis Toolkit (KAT)(Mapleson et al. 2017).

**Supplementary Note 1.6: Generation of simulated data**

The simulated dataset was generated using pIRS (profile-based Illumina pair-end reads simulator)(Hu et al. 2012) which allows the simulation of single-nucleotide variants (SNVs), indels and structural variants (SVs) based on a reference genome in fasta format. The output genome (fasta) can then be used as input to simulate further mutational events at another ‘time point’. Each fasta file is then used as a basis to simulate whole-genome sequencing to produce fastq files with error and quality distributions and coverage bias matching what is seen in real sequencing data. First, a ‘Normal’ file (N) was generated using human reference genome hg38 and a coverage of 5x. Next, SNVs, indels and SVs are introduced to create a file representing cancer, ‘C’, which was then ‘mutated’ 3 different times to produce 3 new files which would represent a more heterogeneous instance of C (C1, C2, C3) each of these were iteratively mutated 2 more times to form evolutionary branches/tumour regions (a, b and c). A second dataset was created using XS(Pratas et al. 2014) and fastx-mutate-tools (<https://github.com/nicolaprezza/fastx-mutate-tools>), of the same format, which instead introduces only SNVs and indels and can produce small scale files (~3GB).

**Supplementary Note 1.7: Analysis of branching pattern using FastGT**

FastGT(Pajuste et al. 2017), an alignment-free approach for calling common SNVs from raw sequencing data, was used to build counts for all samples attributed to patient P90 based on SNVs defined in FastGT’s EX_1.0 database designed for WES data. A variance filter was then used with a cut-off of 0.8 to remove SNV regions unlikely to be contributing to the branching seen. Finally, t-tests (package: genefilter) were performed in R using groups defined by the branching pattern produced by NUQA: Initial A, B, E vs Initial C, D, F; Initial A, B, E vs Recurrence 1A, 1B and Initial C, D, F vs Recurrence 1A, 1B.

**2. Identifying optimal parameters**

**
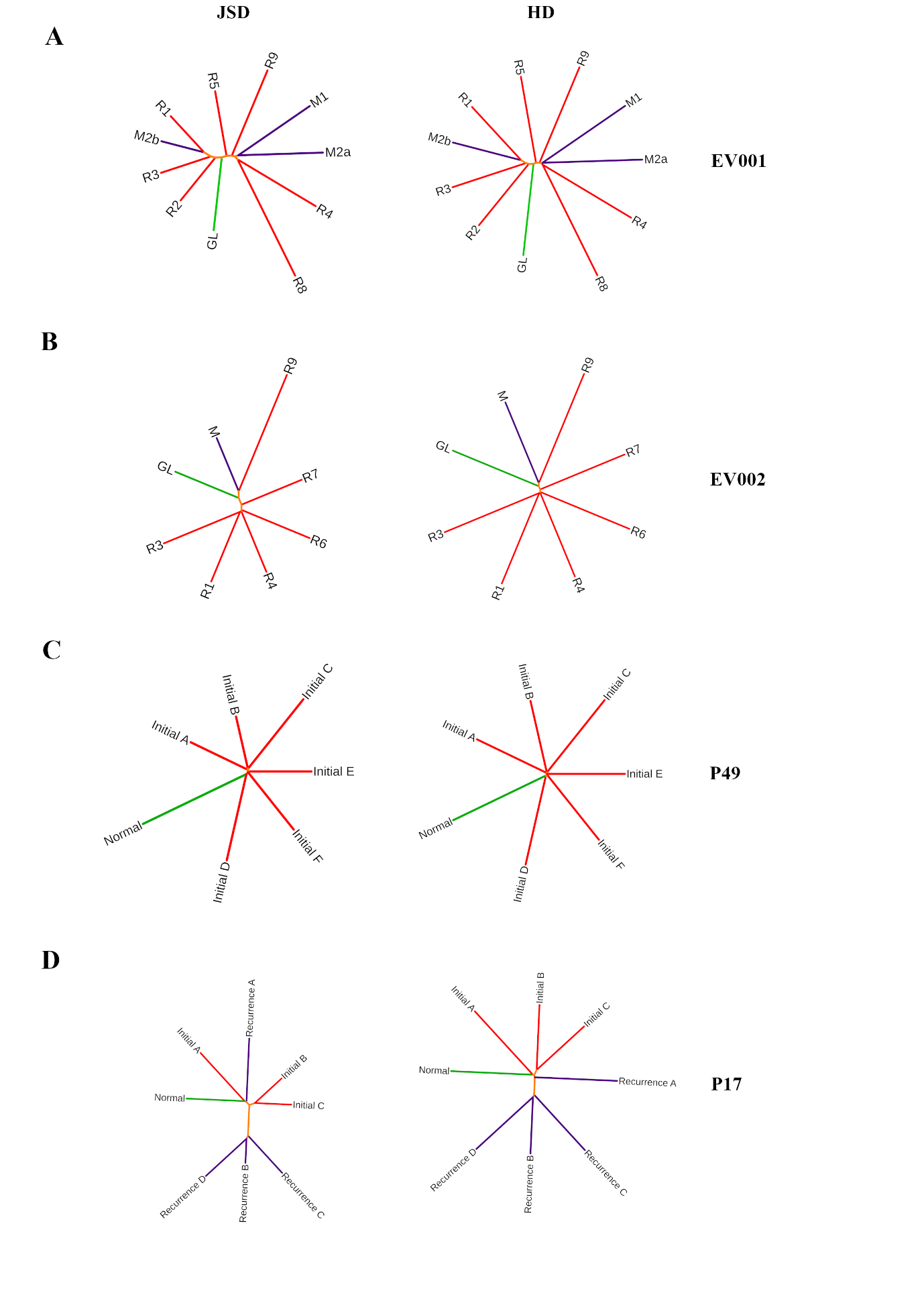
**

**Supplementary Figure S2: Jensen-Shannon Divergence (JSD) and Hellinger Distance (HD) applied as part of an alignment-free algorithm to longitudinal cancer patient cohorts of exome-seq data**. Application of JSD and HD to (**A**) ccRCC patient EV001 with a germ-line sample (GL), multiple samples from the ccRCC tumour (R1-5, R8, R9), one sample from a metastatic tumour (M1) and two samples from a second metastatic tumour (M2a and M2b), (**B**) ccRCC patient EV002 with a germ-line sample (GL), multiple samples from the ccRCC tumour (R1, R3, R4, R6, R7 and R9) and one sample from a metastatic tumour (M), (**C**) glioma patient P49 with a germ-line sample (Normal) and six samples from the initial grade II glioma (Initial A-F) and (**D**) glioma patient P17 with a germ-line sample (Normal), three samples from the initial grade II glioma (Initial A-C) and 2 samples from a recurrent grade IV glioma (Recur A-D).

**
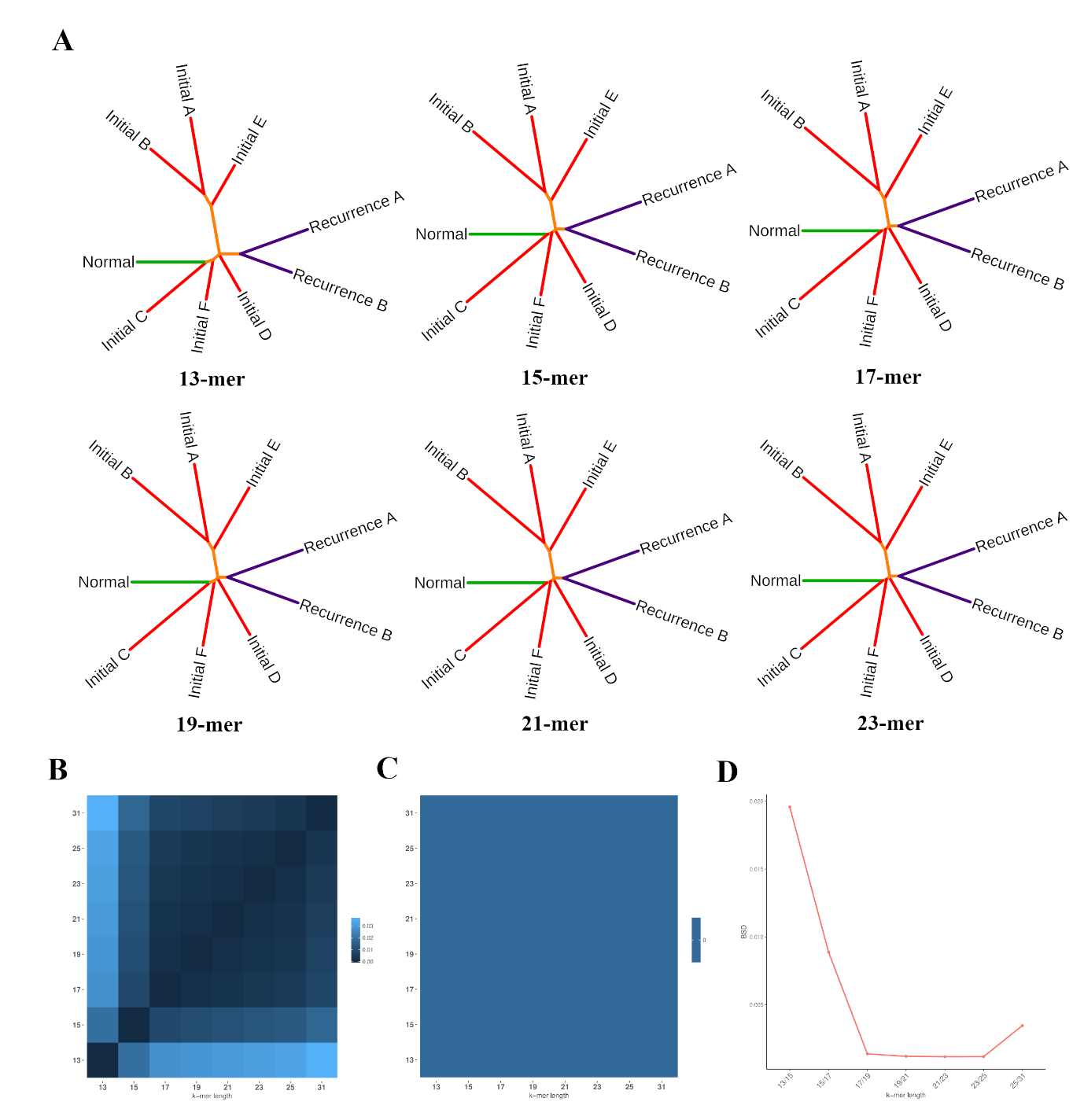
**

**Supplementary Figure S3: Identification of the optimal *k*-mer length for application of AF methods to cancer patient cohorts using patient P90**. (**A**) Tree topologies produced using *k*-mer lengths 13, 15, 17, 19, 21 and 23 in combination with JSD when applying AF methods to patient P90. (**B**) A heatmap representing the Branch-Score distance (BSD) between trees produced using varying *k*-mer lengths (13-31) and HD applied to patient P90. (**C**) A heatmap representing the BSD between trees produced using varying *k*-mer lengths and JSD applied to patient P90. (**D**) A line graph representing the BSD between trees produced using increasing *k*-mer lengths for HD and JSD.


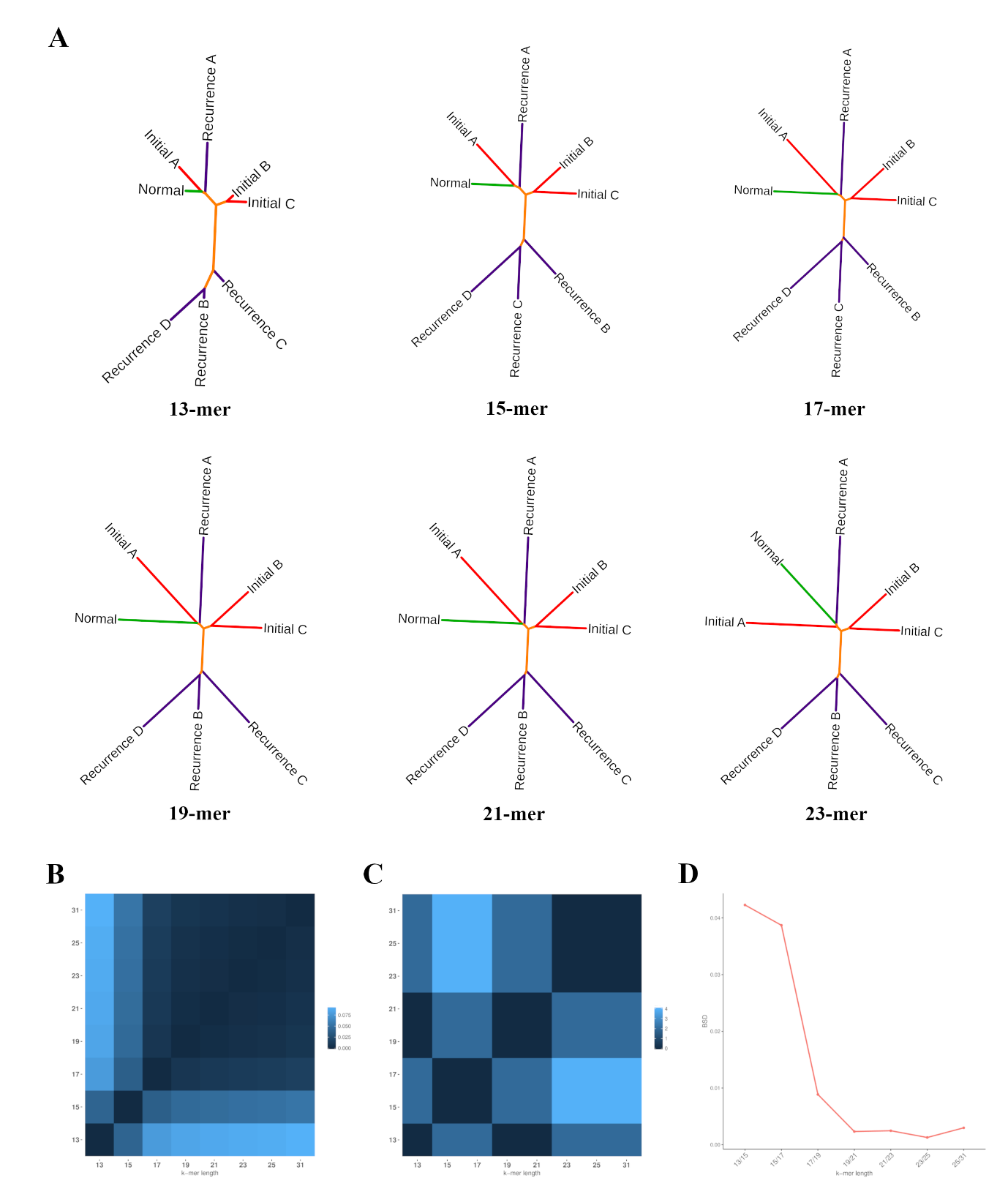


**Supplementary Figure S4: Identification of the optimal k-mer length for application of AF methods to cancer patient cohorts using patient P17.** (**A**) Tree topologies produced using k-mer lengths 13, 15, 17, 19, 21 and 23 in combination with JSD (**B**) A heatmap representing the Branch-Score distance (BSD) between trees produced using varying k-mer lengths (13-31) and JSD. (**C**) A heatmap representing the Symmetric distance between trees produced using varying k-mer lengths and JSD. (**D**) A line graph representing the BSD between trees produced using increasing k-mer lengths for JSD.

**
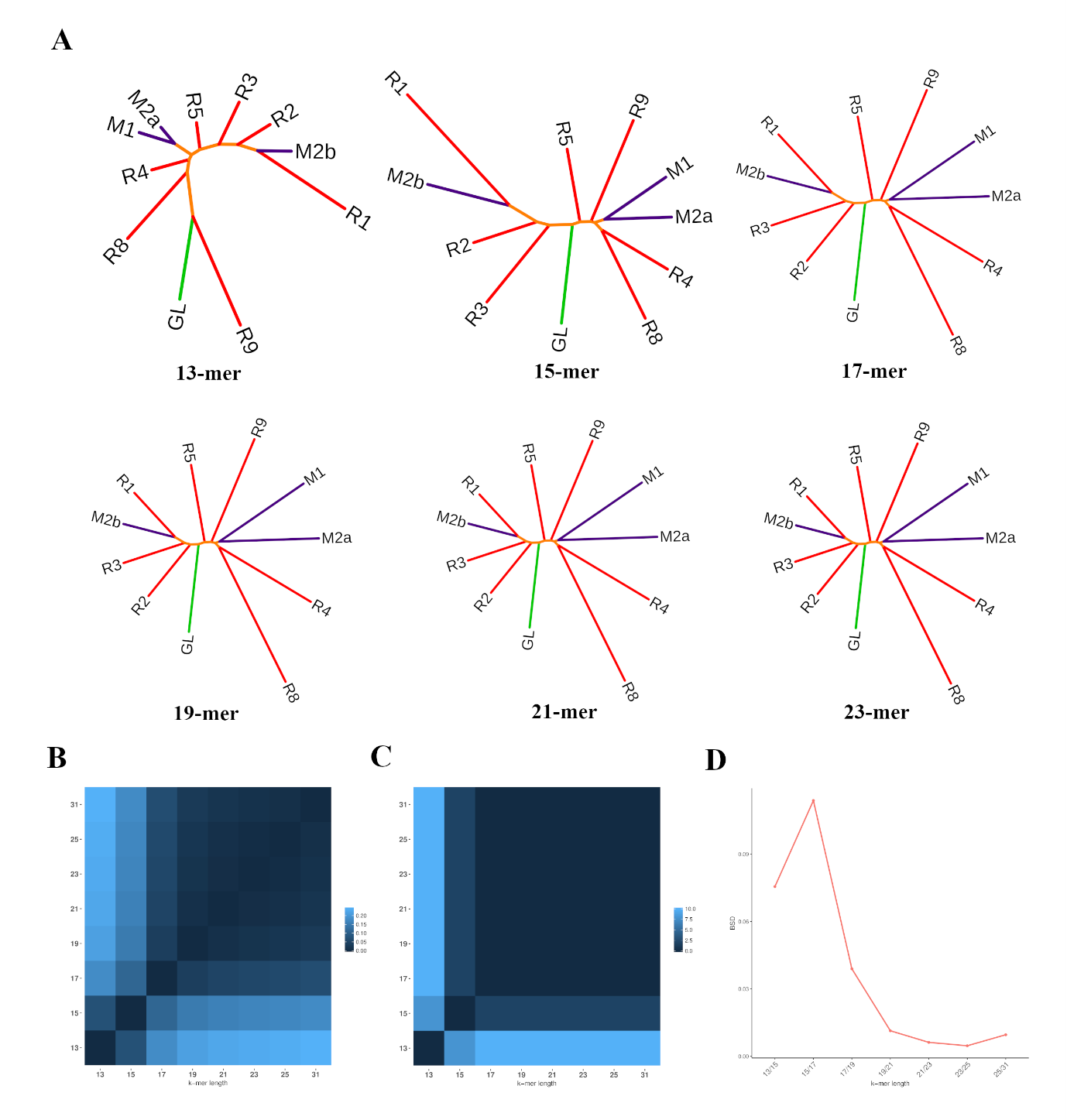
**

**Supplementary Figure S5: Identification of the optimal k-mer length for application of AF methods to cancer patient cohorts using patient EV001.** (**A**) Tree topologies produced using k-mer lengths 13, 15, 17, 19, 21 and 23 in combination with JSD (**B**) A heatmap representing the Branch-Score distance (BSD) between trees produced using varying k-mer lengths (13-31) and JSD. (**C**) A heatmap representing the Symmetric distance between trees produced using varying k-mer lengths and JSD. (**D**) A line graph representing the BSD between trees produced using increasing k-mer lengths for JSD

**
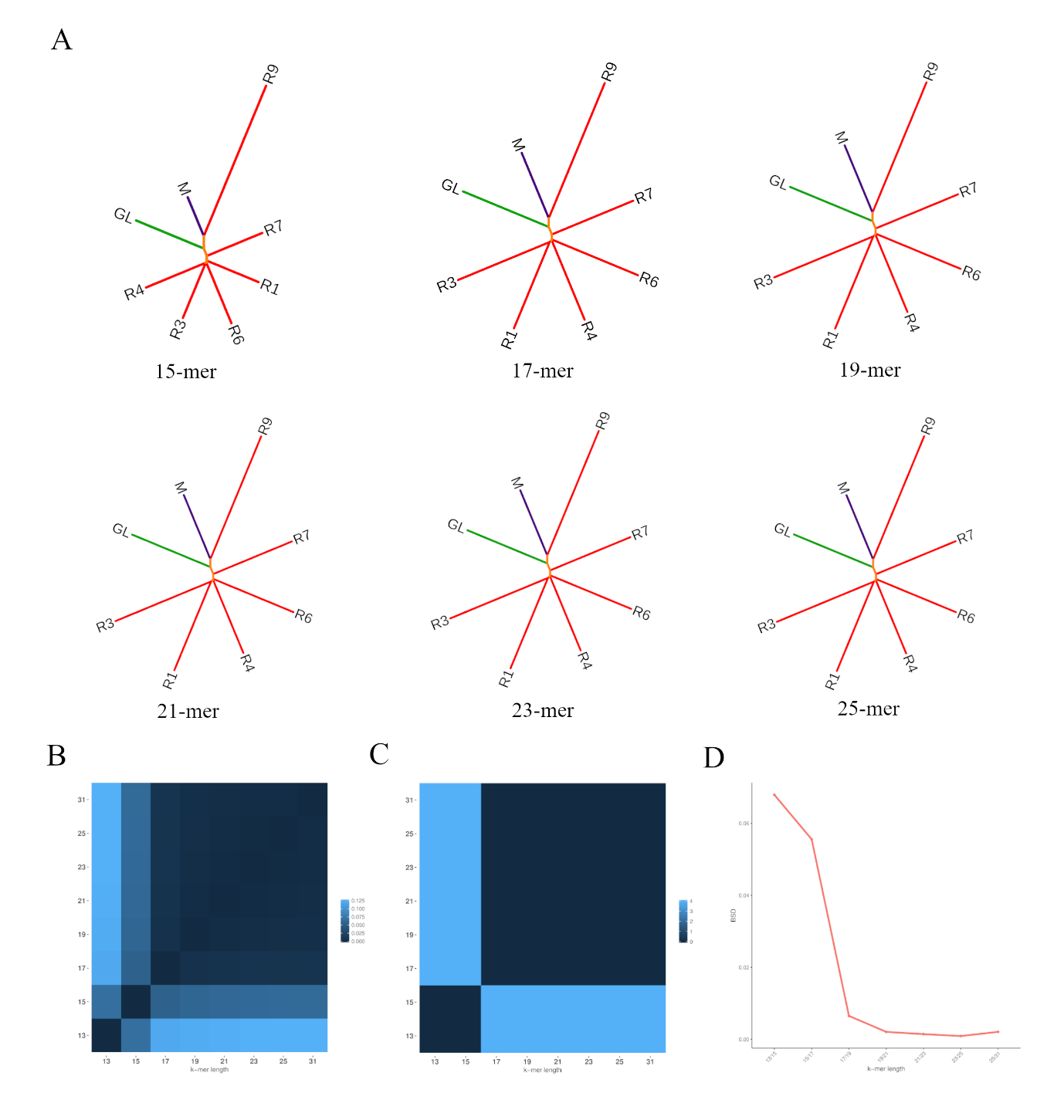
**

**Supplementary Figure S6: Identification of the optimal k-mer length for application of AF methods to cancer patient cohorts using patient EV002.** (**A**) Tree topologies produced using k-mer lengths 15, 17, 19, 21, 23 and 25 in combination with JSD (**B**) A heatmap representing the Branch-Score distance (BSD) between trees produced using varying k-mer lengths(13-31) and JSD. (**C**) A heatmap representing the Symmetric distance between trees produced using varying k-mer lengths and JSD. (**D**) A line graph representing the BSD between trees produced using increasing k-mer lengths for JSD

**3. Application to cancer patient cohorts**


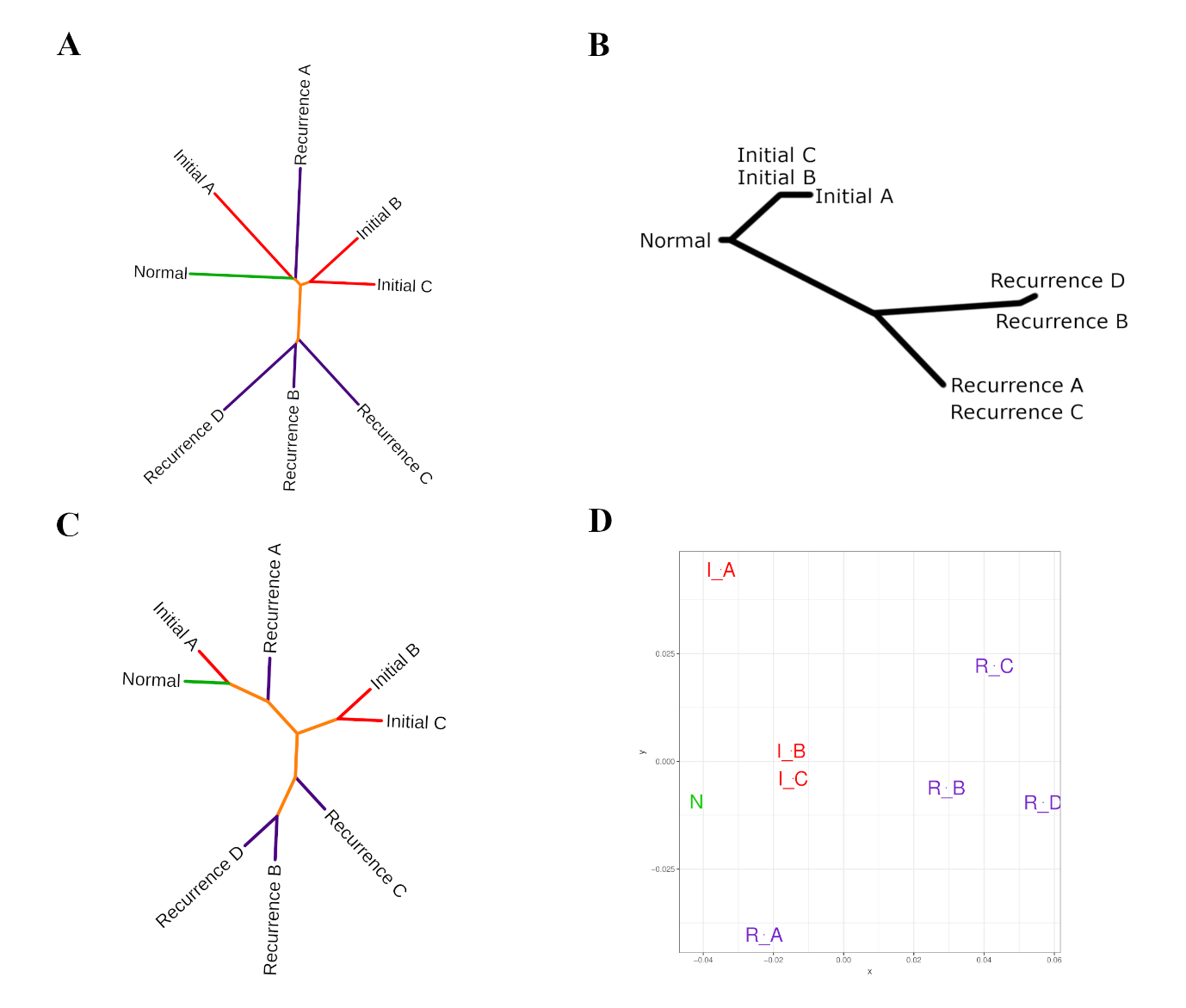


**Supplementary Figure S7: Applying alignment-free sequence comparison methods to glioma patient P17**. (**A**) An unrooted neighbour-joining tree produced applying our alignment-free algorithm, incorporating JSD, to patient P17. (**B**) Least-square minimum-evolution tree produced based on a binary matrix of SNVs present in the samples adapted from Mazor *et al*. (**C**) An unrooted neighbour-joining tree produced applying our AF algorithm, incorporating JSD, to patient P17 ignoring branch length values. (**D**) Multi-dimensional scaling plot representing the distances between samples produced through our AF approach, incorporating JSD, applied to patient P17.

**Supplementary Note 3.1: Interpretation of application of alignment-free to P17**

Patient P17 from the glioma cohort consisted of spatial and temporal samples from whole-exome sequenced (WES) approaches including a circulating blood sample (Normal), three samples from the initial tumour (Initial A, Initial B and Initial C) classified as a grade II glioma and 4 samples from a recurrence tumour (Recurrence A, Recurrence B, Recurrence C and Recurrence D) classified as a grade IV glioblastoma. We applied NUQA to this patient collection and produced phylogenetic trees and MDS plots based on the resultant output (Figure 1B and C). To produce a phylogenetic tree from the WES data, Mazor *et al.* aligned reads and called variants to identify SNVs and indels with only validated protein-coding or splice-site mutations being used in further analysis(Mazor et al. 2015). A binary matrix was created from this with normal samples containing none of the mutations. Manhattan’s distance was used to produce a distance matrix which, in turn, was used to create a least-squares minimum evolution (LSME) tree (Figure S6B). We use this as a basis for comparison, aware that restrictions will have been introduced as only reads which uniquely aligned to the reference genome have been considered and the variant callers used could only identify SNVs and small in/dels but not larger aberrations. This tree suggests that genetic divergence between the initial and recurrent tumours occurs early in evolution indicating branched evolution. Kim *et al.* have previously described this pattern of evolution occurring in glioblastoma tumours which have recurred at a distal site within the brain compared to the initial tumour, however, in this case the tumour recurred close to the original site(Kim et al. 2015). In contrast, the tree produced using NUQA suggests a more linear evolutionary pathway with 'Recurrence A' branching very closely with the initial samples (Figure S6A). Additionally, the LSME tree suggests that there is little ITH within the initial tumour while the recurrent tumour seems to have increased levels of ITH with 2 distinct subclones forming, one containing Recur A and C and the other containing Recur B and D. Similarly, the phylogenetic tree produced using NUQA portrays that the initial tumour is genetically very similar to the Normal sample, with little ITH being displayed within the initial tumour. These patterns can also easily be seen when visualising the distance matrix as an MDS plot (Figure S6D). However, while large levels of ITH are still being seen within the recurrent samples, the tree produced using NUQA suggests that samples B, C and D are more genetically divergent from sample A than perhaps previously understood.

**
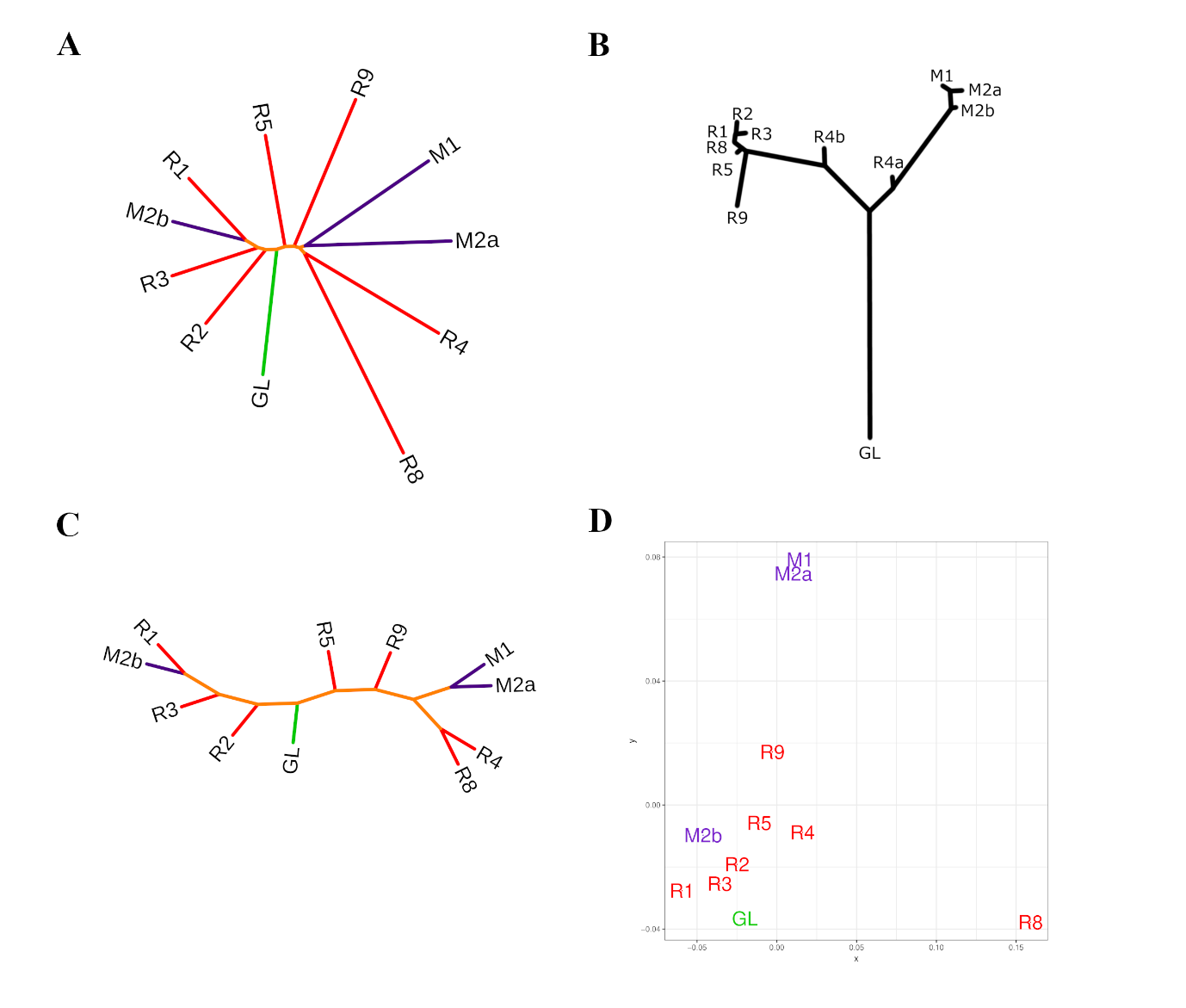
**

**Supplementary Figure S8: Applying alignment-free sequence comparison methods to ccRCC patient EV001**. (**A**) An unrooted neighbour-joining tree produced applying our AF algorithm, incorporating JSD, to patient EV001. (**B**) A maximum parsimony tree produced based on a binary matrix of SNVs present in the samples adapted from Gerlinger *et al*. (**C**) An unrooted neighbour-joining tree produced applying our AF algorithm, incorporating JSD, to patient EV001 ignoring branch length values. (**D**) Multi-dimensional scaling plot representing the distances between samples produced through our AF approach, incorporating JSD, applied to patient EV001.

**Supplementary Note 3.2: Interpretation of application of alignment-free to EV001**

CcRCC patient EV001 had longitudinal, Exome-seq data for germline DNA in the blood (GL), 7 samples from the initial ccRCC tumour (R1-5, R8 and R9) and 3 samples from two metastatic tumours (M1, M2a and M2b). We produced phylogenetic trees and MDS plots based on our output from NUQA for this patient cohort (Figure S7). To produce the original maximum parsimony trees, reads were aligned and variants were called to identify non-synonymous somatic mutations which were then validated and loaded into a binary matrix as described in the original paper (Figure S7B)(Gerlinger et al. 2014). This highlighted only SNVs and small in/dels found to be present within the tumour samples. The original maximum parsimony tree suggests branched evolution with two distinct branches, one containing primarily primary samples and the second containing metastatic samples and sample R4. This suggests that the tumour developed metastatic potential early in evolution. Similar patterns can be seen within the tree produced using NUQA where samples R1-3 are clustering closely and samples R5, R8 and R9 are also clustering together. Additionally, the metastatic samples all appear to occur late in evolution and are the most distal from the germ-line sample. However, there are fundamental differences in that two distinct branches form, one containing M2b and the other containing M1 and M2a. This suggests that the primary and metastatic tumours may be more closely related than originally thought and that greater aberrations are at play than what is portrayed in the original maximum parsimony trees.

**Supplementary Note 3.3: A demonstration of methods to identify potential genomic contributions to the branching pattern obtained through NUQA**

Using FastGT to assign counts values for both reference and alternative alleles of a range of common SNVs found in WES data for each sample attributed to patient P90 gives the potential to identify SNVs and the genotype of these variants therefore also giving the potential to highlight copy-number alterations. Using the tree produced through NUQA (Figure 2C) three groups were identified: Initial A, B and E (ABE); Initial C, D and F(CDF) and Recurrence 1A and 1B (R). These groups then defined contrasts which were assessed using ‘rowttests’ (genefilter) in R Statistical environment for both reference and alternative alleles individually (Table 3.1 and 3.2, respectively). Interestingly, the top gene reported in the contrast ABE and CDF on the reference allele is BRCA2, which has recently been suggested as a molecular target which may sensitize glioma to alkylating agents such as temozolomide, commonly used to treat glioma (Quiros et al. 2011; Chai et al. 2015). In addition, the top gene reported in contrast CDF vs R on the alternative allele is KRAS an oncogene known to be involved in gliomagenesis. This analysis has returned interesting results however, further validation is beyond the scope of this manuscript.

| **ABE vs CDF** | | **ABE vs R** | | **CDF vs R** | |
| --- | --- | --- | --- | --- | --- |
| **SNP identifier** | **Gene** | **SNP identifier** | **Gene** | **SNP identifier** | **Gene** |
| rs587780531 | BRCA2 | rs17851001 | COQ8B | rs199473314 | SCN5A |
| rs371842822 | FGB | rs778173280 | NDUFA10 | rs368727585 | UBA1 |
| rs371123633 | GJB6 | rs143054357 | VWF | rs371891414 | SCN5A |
| rs3742290 | ALG11,UTP14C | rs5352 | EDNRB-AS1,EDNRB | rs760272304 | BRCA2 |
| rs116653247 | FREM2 | rs773075581 | POLD1 | rs150930758 | HERC2 |
| rs558390128 | LPL | rs372028373 | VWF | rs77122016 | SLC12A6 |
| rs111797345 | LMOD3 | rs782047045 | HCFC1 | rs11548543 | EIF2S3 |
| rs902321 | SUCLG2 | rs138675304 | CENPJ | rs765654561 | OPHN1 |
| rs5352 | EDNRB-AS1,EDNRB | rs143380072 | ABCB7 | rs775543440 | FBN1 |
| rs78628025 | CENPJ | rs782376691 | NSDHL | rs5352 | EDNRB-AS1,EDNRB |
| rs148158093 | RPL36A-HNRNPH2,GLA | rs758218321 | PHYH | rs748362699 | HERC2 |
| rs752516966 | GPC3 | rs148229804 | WRN | rs104893743 | HYAL1 |
| rs149382837 | FPR1 | rs200865354 | CACNA1C-AS1,CACNA1C | rs35468447 | MEGF8 |
| rs76434661 | GJB2 | rs762656408 | CACNA1C | rs138177581 | HERC2 |
| rs55709737 | PRKCD | rs35977912 | AGXT | rs148743497 | ABHD5 |

**Table 3.1:** Top 15 genes identified for each of 3 contrasts (ABE vs CDF, ABE vs R and CDF vs R) comparing SNP counts identified on the reference allele using FastGT, identified as significant using t-tests (p<0.01).

| **ABE vs CDF** | | **ABE vs R** | | **CDF vs R** | |
| --- | --- | --- | --- | --- | --- |
| **SNP identifier** | **Gene** | **SNP identifier** | **Gene** | **SNP identifier** | **Gene** |
| rs626169 | CTDP1 | rs2737699 | SACS | rs4362222 | KRAS |
| rs2227291 | ATP7A | rs56223054 | TBC1D4 | rs1126670 | ADH4 |
| rs2737699 | SACS | rs34244923 | TBX22 | rs141225099 | MLC1 |
| rs6491066 | ATP8A2 | rs1522384 | FLNB | rs17863783 | UGT1A*** |
| rs1131665 | IRF7 | rs114814153 | SLC6A4 | rs1131265 | TIMMDC1 |
| rs34436714 | NLRP12 | rs149854452 | SLC6A20 | rs1131265 | TMEM39A1, TIMMDC1 |
| rs12459008 | ZNF816A | rs63751001 | ABCC6 | rs8140207 | TRIOBP |
| rs376936511 | WDR45 | rs2230469 | PIP4K2A | rs3759871 | SPG11 |
| rs5977625 | FRMD7 | rs62144168 | PRPF31 | rs769267 | NR |
| rs17078605 | SACS | rs17078605 | SACS | rs3194051 | IL7R |
| rs3617 | BAP1* | rs12874397 | FREM2 | rs7300444 | WNK1 |
| rs3617 | NR | rs6491066 | ATP8A2 | rs17053501 | CACNA1D |
| rs3617 | ITIH3 | rs4362222 | KRAS | rs4988958 | IL1Rl1, IL1RL2, IL18R1 |
| rs3617 | NT5DC2** | rs12459008 | ZNF816A | rs3764021 | NR |
| rs4462937 | DNAH12 | rs5956583 | XIAP | rs1494558 | IL7R |

**Table 3.2:** Top 15 genes identified for each of 3 contrasts (ABE vs CDF, ABE vs R and CDF vs R) comparing SNP counts identified on the alternative allele using FastGT, identified as significant using t-tests (p<0.01).

* Additional names: DNAH1,GLT8D1,GLYCTK,GLYCTK-AS1,GNL3,ITIH1,ITIH3,ITIH4,ITIH4-AS1,MIR135A1,MIR8064,MIRLET7G,MUSTN1,NEK4,NISCH,NT5DC2,PBRM1,PHF7,PPM1M,SEMA3G,SFMBT1,SMIM4,SNORD136,SNORD19,SNORD19B,SNORD69,SPCS1,STAB1,TLR9,TMEM110,TMEM110-MUSTN1,TNNC1,TWF2,WDR82

** Additional names: PBRM1,GLT8D1,GNL3,ITIH1,ITIH3,ITIH4,MUSTN1,NEK4,NISCH,SMIM4,SPCS1, STAB1, TMEM110, TMEM110-MUSTN1

*** Additional names: UGT1A10,UGT1A6,UGT1A7,UGT1A8,UGT1A9

**4. Benchmarking NUQA**

**Supplementary Note 4.1: The effect of alignment on alignment-free phylogenetic analysis using simulated data containing structural variation**

In order to assess the effect of conventional SNP alignment towards structural variation through the output produced by NUQA, a tree was produced using a simulated set where structural variation was introduced at a rate of 0.01-0.1% to WGS data using pIRS (Hu et al. 2012). A second tree was produced using only aligned reads (BWA version 0.7.15)(Li and Durbin 2009) on the same data that were reversed from .bam files to .fastq using samtools(Li et al. 2009). We followed a similar approach adopted earlier of creating a reference (‘N’), a perturbed file (‘C’) and three deviations of ‘C’, each deviated itself three times (e.g. C1a, C1b and C1c). This approach has several limitations in that the simulated set is manifested from the same reference genome as it is aligned to (in this case, hg38) therefore not allowing for natural in situ aberrant processes. Also, WGS with shallow depths, although producing large files, may be dominated, via k-mers, by SNP events. This simulated approach, however, means that structural variation should be one of the dominant causes of unaligned-reads. Following alignment, ~1% reads were removed suggesting that structural variations were indeed extracted between the two NUQA trees of the same data allowing us to deduce any alterations. It can be seen from Figure S9A, of the ‘complete’ NUQA tree, that ‘C’ sits in a unique branch and each of the C1’s, C2’s and C3’s branch closely together as expected. However, in the ‘aligned’ tree (Figure S9B), ‘C’ has now moved to be nested within the C1 branching lineage. The removal of some of the unique information has shifted its ability to be uniquely classified.


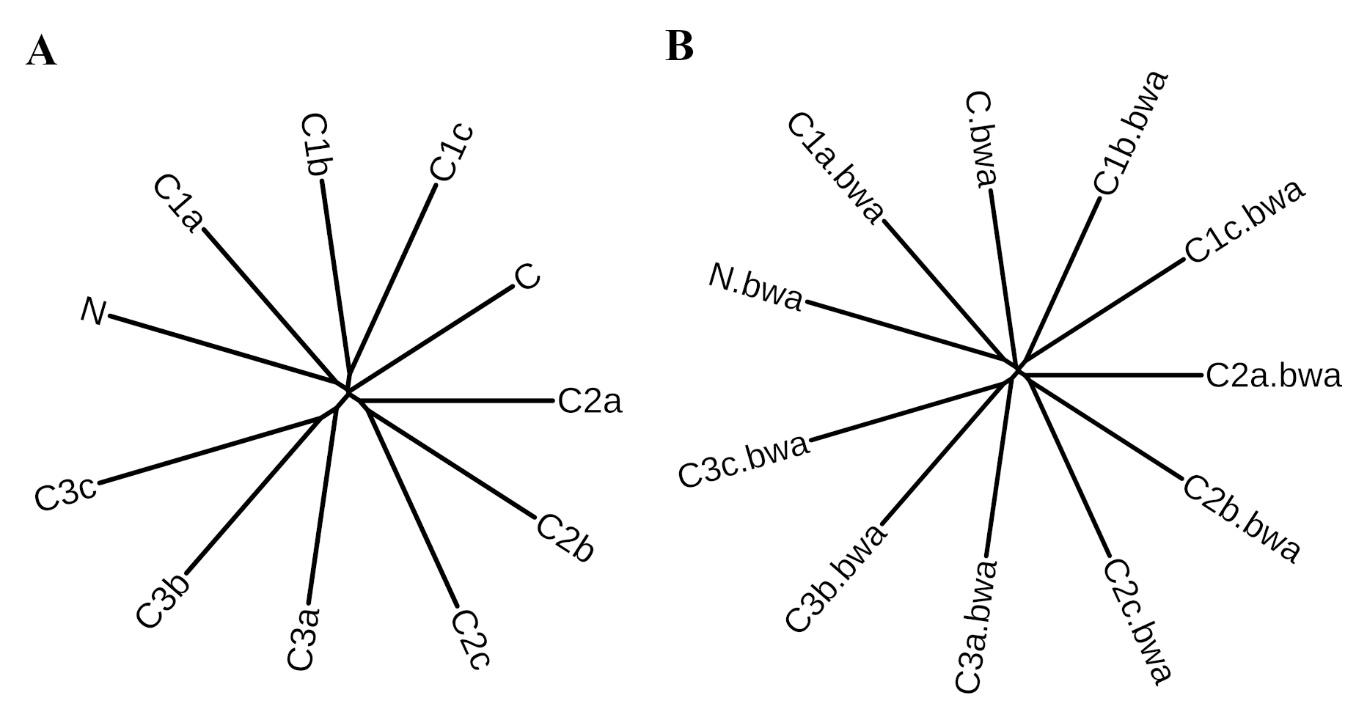


**Supplementary Figure S9: Applying alignment-free sequence comparison methods to an aligned and unaligned simulated dataset**. (**A**) An unrooted neighbour-joining tree produced applying our AF algorithm using JSD and k=21 to a simulated dataset for which SNVs, indels and structural variation has been introduced using the software pIRS. (**B**) An unrooted neighbour-joining tree produced applying our AF algorithm using JSD and k=21 using the same simulated dataset but removing reads which were not aligned when using BWA to align to hg38.

**References**

Aflitos SA, Severing E, Sanchez-Perez G, Peters S, de Jong H, de Ridder D. 2015. Cnidaria: Fast, reference-free clustering of raw and assembled genome and transcriptome NGS data. BMC Bioinformatics. 16(1):1–10. doi:10.1186/s12859-015-0806-7.

Chai KM, Wang C-Y, Liaw H-J, Fang K-M, Yang C-S, Tzeng S-F. 2015. Downregulation of BRCA1-BRCA2-containing complex subunit 3 sensitizes glioma cells to temozolomide. Oncotarget. 5(21). doi:10.18632/oncotarget.2543.

Dai Q, Yang Y, Wang T. 2008. Markov model plus k-word distributions: A synergy that produces novel statistical measures for sequence comparison. Bioinformatics. 24(20):2296–2302. doi:10.1093/bioinformatics/btn436.

Fan H, Ives AR, Surget-Groba Y, Cannon CH. 2015. An assembly and alignment-free method of phylogeny reconstruction from next-generation sequencing data. BMC Genomics. 16(1):1–18. doi:10.1186/s12864-015-1647-5.

Gerlinger M, Horswell S, Larkin J, Rowan AJ, Salm MP, Varela I, Fisher R, Mcgranahan N, Matthews N, Santos CR, et al. 2014. Articles Genomic architecture and evolution of clear cell renal cell carcinomas defined by multiregion sequencing. Nat Publ Gr. 46(3):225–233. doi:10.1038/ng.2891.

Höhl M, Rigoutsos I, Ragan MA. 2007. Pattern-based phylogenetic distance estimation and tree reconstruction. Evol Bioinform Online. 2(2003):359–75. doi:10.1080/10635150701294741.

Hu X, Yuan J, Shi Y, Lu J, Liu B, Li Z, Chen Y, Mu D, Zhang H, Li N, et al. 2012. pIRS: Profile-based illumina pair-end reads simulator. Bioinformatics. 28(11):1533–1535. doi:10.1093/bioinformatics/bts187.

Kim J, Lee IH, Cho HJ, Park CK, Jung YS, Kim Y, Nam SH, Kim BS, Johnson MD, Kong DS, et al. 2015. Spatiotemporal Evolution of the Primary Glioblastoma Genome. Cancer Cell. 28(3):318–328. doi:10.1016/j.ccell.2015.07.013.

Li H, Durbin R. 2009. Fast and accurate short read alignment with Burrows-Wheeler transform. Bioinformatics. 25(14):1754–60. doi:10.1093/bioinformatics/btp324.

Li H, Handsaker B, Wysoker A, Fennell T, Ruan J, Homer N, Marth G, Abecasis G, Durbin R. 2009. The Sequence Alignment/Map format and SAMtools. Bioinformatics. 25(16):2078–2079. doi:10.1093/bioinformatics/btp352.

Mapleson D, Accinelli GG, Kettleborough G, Wright J, Clavijo BJ. 2017. KAT: A K-mer analysis toolkit to quality control NGS datasets and genome assemblies. Bioinformatics. 33(4):574–576. doi:10.1093/bioinformatics/btw663.

Marçais G, Kingsford C. 2011. A fast, lock-free approach for efficient parallel counting of occurrences of k-mers. Bioinformatics. 27(6):764–770. doi:10.1093/bioinformatics/btr011.

Mazor T, Pankov A, Johnson BE, Hong C, Hamilton EG, Bell RJA, Smirnov I V., Reis GF, Phillips JJ, Barnes MJ, et al. 2015. DNA Methylation and Somatic Mutations Converge on the Cell Cycle and Define Similar Evolutionary Histories in Brain Tumors. Cancer Cell. 28(3):307–317. doi:10.1016/j.ccell.2015.07.012.

Mckenna A, Hanna M, Banks E, Sivachenko A, Cibulskis K, Kernytsky A, Garimella K, Altshuler D, Gabriel S, Daly M, et al. 2010. The Genome Analysis Toolkit : A MapReduce framework for analyzing next-generation DNA sequencing data. :1297–1303. doi:10.1101/gr.107524.110.20.

Pajuste FD, Kaplinski L, Möls M, Puurand T, Lepamets M, Remm M. 2017. FastGT: An alignment-free method for calling common SNVs directly from raw sequencing reads. Sci Rep. 7(1):1–10. doi:10.1038/s41598-017-02487-5.

Picard Toolkit. 2019. Broad Institute, GitHub Repos.

Pratas D, Pinho AJ, Rodrigues JMOS. 2014. XS: A FASTQ read simulator. BMC Res Notes. 7(1). doi:10.1186/1756-0500-7-40.

Quiros S, Roos WP, Kaina B. 2011. Rad51 and BRCA2 - new molecular targets for sensitizing glioma cells to alkylating anticancer drugs. PLoS One. 6(11). doi:10.1371/journal.pone.0027183.

Sims GE, Jun S-R, Wu GA, Kim S-H. 2009. Alignment-free genome comparison with feature frequency profiles (FFP) and optimal resolutions. Proc Natl Acad Sci. 106(8):2677–2682. doi:10.1073/pnas.0813249106.

Vinga S, Gouveia-Oliveira R, Almeida JS. 2004. Comparative evaluation of word composition distances for the recognition of SCOP relationships. Bioinformatics. 20(2):206–215. doi:10.1093/bioinformatics/btg392.

Zielezinski A, Vinga S, Almeida J, Karlowski WM. 2017. Alignment-free sequence comparison: Benefits, applications, and tools. Genome Biol. 18(1):1–17. doi:10.1186/s13059-017-1319-7.
